# Delayed Vesicular Urticarial Dermatosis Due to Apixaban

**DOI:** 10.7759/cureus.17171

**Published:** 2021-08-14

**Authors:** Wendy T Garzon-Siatoya, Dan Morgenstern-Kaplan, Karol Avila-Castano, Masih Rezaee, Alexei Gonzalez-Estrada

**Affiliations:** 1 Division of Pulmonary, Allergy, and Sleep Medicine, Department of Medicine, Mayo Clinic, Jacksonville, USA

**Keywords:** apixaban, adverse drug reaction, delayed hypersensitivity, drug challenge, skin testing, patch testing

## Abstract

Cutaneous adverse drug reactions (cADR) are delayed hypersensitivity reactions that are T-cell mediated. Novel oral anticoagulants, including Factor Xa inhibitors, are increasingly used as therapeutic or prophylactic management of thrombosis and atrial fibrillation.

We introduce the case of a 78-year-old woman with no known allergies and a history of atrial fibrillation who was started on apixaban for cerebrovascular accident prophylaxis. Approximately nine days after starting apixaban, she developed a vesicular-urticated erythematous rash initially located on her right upper extremity, progressing to her face. She was evaluated after two weeks for the persistence of symptoms and improved with hydroxyzine and prednisone. Subsequently, she was advised to discontinue and evade all Factor Xa inhibitors, including apixaban, and was switched to warfarin.

Naranjo score scale was 5-6. The patient declined skin biopsy and drug challenge. After a month of discontinuation of systemic steroids, patch testing was performed with apixaban, rivaroxaban, and edoxaban, with a negative result. Since this episode, the patient has not had a recurrence of the rash.

As far as we know, this is the first case report of a non-severe cADR to apixaban. Even though there are no standardized protocols for diagnosing drug reactions to Factor Xa inhibitors, patch testing at increasing non-irritant concentrations with re-challenge of alternative agents and the suspected offending agent, if possible, should be included in the evaluation of a cADR.

## Introduction

Cutaneous adverse drug reactions (cADR) are delayed hypersensitivity reactions mediated by T-cells and represent the most common type of adverse drug reaction (ADR) [1]. Antibiotics are the most common type of medication known to cause cADR, while anticonvulsants and antipyretics are the second and third most common causes of cARD, respectively [1].

Novel oral anticoagulants (NOACs) are prescribed at an increasing frequency for cerebrovascular accident (CVA) prevention in patients with non-valvular atrial fibrillation. Factor Xa inhibitors are a group of NOACs which include apixaban and rivaroxaban, among other drugs. ADRs from apixaban include an increased risk of bleeding, thrombocytopenia, and nausea. In rare cases, severe reactions can occur, including vasculitis and skin necrosis [2-4]. To date, there are no reported cases of non-severe cADR due to apixaban. This article was previously presented as a meeting abstract at the American College of Allergy, Asthma, and Immunology 2017 Annual Scientific Meeting on October 26-30, 2017.

## Case presentation

A 78-year-old woman with a medical history of cardiovascular disease, hypertension, and atrial fibrillation, and CHA2DS2VASc score of 6 (congestive heart failure=1, hypertension=1, age>75=2, diabetes=1, stroke or transient ischemic attack=2, vascular disease=1, age 65-74=1, sex category/female=1) was evaluated for a pruritic rash of two weeks of evolution. She first noticed the rash nine days after starting apixaban, indicated for CVA prophylaxis in the setting of atrial fibrillation. The rash initially appeared on her right upper extremity, progressing to her face, and was not related to any other symptoms. She was not on any other new medications other than apixaban. She had no known medication or other allergies and had never had a similar rash.

**Figure 1:**
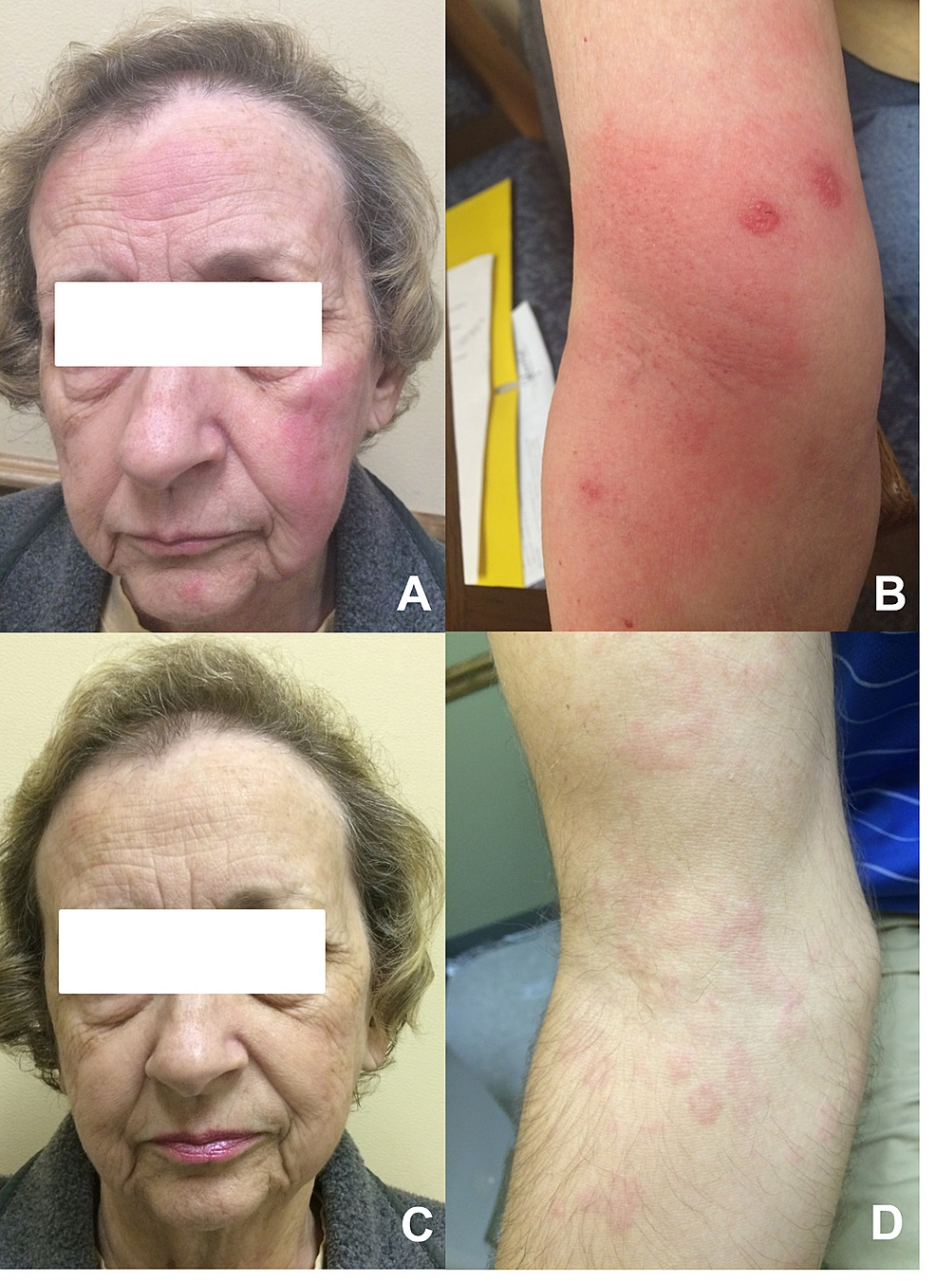
Clinical presentation of the patient (A, B) Vesicular urticarial dermatosis of the face and right antecubital fossa with three clusters of vesicles developed during treatment with apixaban. (C, D) Five days after the discontinuation of apixaban and treatment with systemic steroids and an antihistaminic.

Her physical exam revealed a vesicular-urticated erythematous rash on the face and right antecubital region with three clusters of vesicles (Figures 1A, 1B). Laboratory evaluation included a complete blood count and comprehensive metabolic panel, which were both within normal limits. The patient declined skin biopsy. Naranjo score scale was 5-6, suggesting probable cADR. She was instructed to discontinue and avoid all Factor Xa inhibitors, including apixaban. The patient was started on warfarin for CVA prophylaxis in the setting of atrial fibrillation and placed on systemic corticosteroids (prednisone 40 mg daily for 10 days) and hydroxyzine 10 mg at bedtime for 10 days. There was a significant improvement of the rash within five days of this intervention (Figures 1C, 1D). After a month of discontinuation of systemic steroids, patch testing was performed with apixaban, rivaroxaban, and edoxaban at 10% and 30% concentration each with petrolatum. Readings at 48 hours, 72 hours, and 96 hours were negative. The patient declined an oral challenge to alternative Factor Xa inhibitors. Since this episode, the patient has not had a recurrence of the rash.

## Discussion

We presented an unusual case of cADR to apixaban in a 78-year-old female patient. Evidence suggests that ADRs are more common among Caucasians and the elderly, with a mean age of approximately 75 years old [5]. In addition, females often tend to have higher rates of ADRs than males [6]. Diagnosis of a medication-induced cADR can be made upon using the Naranjo ADR probability scale, which assesses the causality of exposure to a drug and the subsequent cADR [7]. The scoring system is based on answering 10 questions about the timing of the development of the cADR and exposure to the suspected offending medication [7].

There are seldom cases of severe cutaneous adverse reaction to apixaban, including leukocytoclastic vasculitis [2,3] and skin necrosis [4] reported in the literature. Currently, there are no reports of non-severe cADR secondary to apixaban. On the contrary, several cases of non-severe cADR to rivaroxaban have been documented [8-10]. For example, one case reported a cADR to rivaroxaban, which resolved after discontinuation of the medication and did not recur when the patient was started on apixaban, suggesting that the allergic reaction was specific to rivaroxaban without a cross-reaction to apixaban [9].

There is no standardized intradermal skin testing or patch testing protocols to diagnose delayed reactions secondary to factor Xa inhibitors [11,12]. Nevertheless, evaluation of a cADR should include patch testing at increasing non-irritant concentrations to all Factor Xa inhibitors with re-challenge of alternative agents after negative patch testing [11].

## Conclusions

Factor Xa inhibitors, such as apixaban and rivaroxaban, are increasingly used to manage thrombosis and as CVA prophylaxis in the presence of atrial fibrillation. Therefore, health care providers must be aware of possible side effects, including cADR. Although few cases of cADR to rivaroxaban are reported in the literature, to the extent of our knowledge, this is the first case report of a non-severe cADR to apixaban. Evaluation of a cADR should include referral to the allergy or dermatology department.

## References

[1] Joint Task Force on Practice Parameters, American Academy of Allergy, Asthma and Immunology, American College of Allergy, Asthma and Immunology, Joint Council of Allergy, Asthma and Immunology (2010). Drug allergy: an updated practice parameter. Ann Allergy Asthma Immunol.

[2] Spears J, Chetrit DA, Manthey S, Lee C, Al-Saiegh Y (2020). Apixaban as a rare cause of leukocytoclastic vasculitis. Case Rep Rheumatol.

[3] Nasir UB, Kumar A, Easwar A (2018). Apixaban causing leukocytoclastic vasculitis. J Allergy Clin Immunol Pract.

[4] Aalaei-Andabili SH, Umer M, Keeley EC (2019). An adult woman with stage 4 chronic kidney disease and a diffuse rash. JAMA Cardiol.

[5] Heng YK, Lim YL (2015). Cutaneous adverse drug reactions in the elderly. Curr Opin Allergy Clin Immunol.

[6] Franconi F, Brunelleschi S, Steardo L, Cuomo V (2007). Gender differences in drug responses. Pharmacol Res.

[7] Naranjo CA, Busto U, Sellers EM (1981). A method for estimating the probability of adverse drug reactions. Clin Pharmacol Ther.

[8] Vernon HM, Nielsen AK, O'Bryan EC (2016). Hypersensitivity reaction after administration of rivaroxaban (Xarelto). Am J Emerg Med.

[9] Sasson E, James M, Russell M, Todorov D, Cohen H (2018). Probable rivaroxaban-induced full body rash: a case report. J Pharm Pract.

[10] Chiasson CO, Canneva A, Roy FO, Doré M (2017). Rivaroxaban-induced hypersensitivity syndrome. Can J Hosp Pharm.

[11] Brockow K, Romano A, Blanca M, Ring J, Pichler W, Demoly P (2002). General considerations for skin test procedures in the diagnosis of drug hypersensitivity. Allergy.

[12] Demoly P, Adkinson NF, Brockow K (2014). International consensus on drug allergy. Allergy.

